# Analgesic Effects of GpTx-1, PF-04856264 and CNV1014802 in a Mouse Model of Na_V_1.7-Mediated Pain

**DOI:** 10.3390/toxins8030078

**Published:** 2016-03-17

**Authors:** Jennifer R. Deuis, Joshua S. Wingerd, Zoltan Winter, Thomas Durek, Zoltan Dekan, Silmara R. Sousa, Katharina Zimmermann, Tali Hoffmann, Christian Weidner, Mohammed A. Nassar, Paul F. Alewood, Richard J. Lewis, Irina Vetter

**Affiliations:** 1 Centre for Pain Research, Institute for Molecular Biosciences, The University of Queensland, St Lucia, QLD 4072, Australia; j.deuis@uq.edu.au (J.R.D.); j.wingerd@imb.uq.edu.au (J.S.W.); t.durek@imb.uq.edu.au (T.D.); z.dekan@imb.uq.edu.au (Z.D.); s.desousa@imb.uq.edu.au (S.R.S.); p.alewood@imb.uq.edu.au (P.F.A.); r.lewis@uq.edu.au (R.J.L.); 2 School of Pharmacy, The University of Queensland, Woolloongabba, QLD 4102, Australia; 3 Department of Physiology and Pathophysiology and Department of Anaesthesiology, Friedrich-Alexander University Erlangen-Nuremberg, 91054 Erlangen, Germany; zoltan.winter@kfa.imed.uni-erlangen.de (Z.W.); katharina.zimmermann@fau.de (K.Z.); tal.hoffmann@fau.de (T.H.); Weidner@physiologie1.uni-erlangen.de (C.W.); 4 Department of Biomedical Science, University of Sheffield, Sheffield S10 2TN, UK; m.nassar@sheffield.ac.uk

**Keywords:** OD1, Na_V_1.7, PF-04856264, CNV1014802, raxatrigine, GpTx-1, pain

## Abstract

Loss-of-function mutations of Na_V_1.7 lead to congenital insensitivity to pain, a rare condition resulting in individuals who are otherwise normal except for the inability to sense pain, making pharmacological inhibition of Na_V_1.7 a promising therapeutic strategy for the treatment of pain. We characterized a novel mouse model of Na_V_1.7-mediated pain based on intraplantar injection of the scorpion toxin OD1, which is suitable for rapid *in vivo* profiling of Na_V_1.7 inhibitors. Intraplantar injection of OD1 caused spontaneous pain behaviors, which were reversed by co-injection with Na_V_1.7 inhibitors and significantly reduced in Na_V_1.7^−/−^ mice. To validate the use of the model for profiling Na_V_1.7 inhibitors, we determined the Na_V_ selectivity and tested the efficacy of the reported Na_V_1.7 inhibitors GpTx-1, PF-04856264 and CNV1014802 (raxatrigine). GpTx-1 selectively inhibited Na_V_1.7 and was effective when co-administered with OD1, but lacked efficacy when delivered systemically. PF-04856264 state-dependently and selectively inhibited Na_V_1.7 and significantly reduced OD1-induced spontaneous pain when delivered locally and systemically. CNV1014802 state-dependently, but non-selectively, inhibited Na_V_ channels and was only effective in the OD1 model when delivered systemically. Our novel model of Na_V_1.7-mediated pain based on intraplantar injection of OD1 is thus suitable for the rapid *in vivo* characterization of the analgesic efficacy of Na_V_1.7 inhibitors.

## 1. Introduction

Nine voltage-gated sodium channel subtypes have been described to date (Na_V_1.1–Na_V_1.9), several of which are implicated as causative contributors to pain. Of particular interest is Na_V_1.7, as loss-of-function mutations in humans lead to congenital insensitivity to pain, a rare condition that results in the inability to sense pain [1,2]. This makes pharmacological inhibition of Na_V_1.7 an exciting therapeutic strategy for the treatment of a wide range of pain types, including inherited erythromelalgia and paroxysmal extreme pain disorder, two conditions whose pathophysiology arises from Na_V_1.7 gain-of-function mutations. Accordingly, the development of Na_V_1.7 inhibitors is being actively pursued by the pharmaceutical industry [3]. Na_V_1.7 selectivity is key to developing more effective analgesics as activity at major off-targets, including the skeletal muscle isoform Na_V_1.4, the cardiac isoform Na_V_1.5, as well as neuronal isoforms Na_V_1.1, Na_V_1.2 and Na_V_1.6, is likely to impact the therapeutic window and cause dose-limiting adverse effects [4,5,6]. However, achieving sufficient selectivity for Na_V_1.7 over the other Na_V_ isoforms has proven challenging due to the high sequence homology within the Na_V_ family [7]. Despite this challenge, several small molecules with reported Na_V_1.7 selectivity are currently undergoing clinical development, including PF-05089771 (Pfizer) and CNV1014802 (Convergence Pharmaceuticals) [8]. In addition to small molecules, venom-derived peptides are proving to be an alternate source for Na_V_1.7 inhibitors, with several spider peptides, including GpTx-1, Huwentoxin-IV and ProTx-II, reported to have some Na_V_1.7 selectivity [9,10,11]. Evaluating the analgesic efficacy of this increasing number of Na_V_1.7-selective compounds in relevant pre-clinical models of pain is a crucial translational step to clinical success. However, the pathophysiological basis of most commonly used animal models is multi-factorial, making selection of the best disease model for rapid efficacy profiling of Na_V_1.7 inhibitors challenging. We thus sought to characterize a novel animal model of Na_V_1.7-mediated pain that is suitable for the rapid *in vivo* profiling of the analgesic efficacy of Na_V_1.7 inhibitors. As gain-of-function mutations of Na_V_1.7 in humans are associated with a range of painful syndromes [12,13], we hypothesized that intraplantar administration of the Na_V_1.7 activator OD1 could be used as a pharmacological tool to establish a Na_V_1.7-mediated mouse model of pain.

OD1 is a scorpion toxin isolated from the venom of the Iranian yellow scorpion (*Odontobuthus doriae*) that potently enhances the activity of Na_V_1.7 by inhibiting channel fast inactivation and increasing peak current with an EC_50_ between 4 and 8 nM [14,15]. OD1 also has activity at Na_V_1.4 (EC_50_ = 10 nM) and Na_V_1.6 (EC_50_ = 30–47 nM); however, it is 1000-fold selective (EC_50_ > 1 μM) over Na_V_1.1, Na_V_1.2, Na_V_1.3, Na_V_1.5 and Na_V_1.8 [14,15]. Consistent with its *in vitro* pharmacological activity, intraplantar administration of OD1 elicited pain behaviors, including licking and flinching of the hind paw, and we have previously used this model to assess the analgesic effects of ProTx-III [16]. The aim of this study was to characterize the OD1 mouse model of pain and to validate the use of this model by testing the efficacy of several reported selective Na_V_1.7 inhibitors, including the spider peptide GpTx-1, PF-04856264 (as the structure of clinical candidate PF-05089771 is not publicly available) [17] and the clinical candidate CNV1014802 (raxatrigine). As the full pharmacological activity of these inhibitors is not reported, we determined their selectivity at Na_V_1.1–Na_V_1.8 and the mode of action at Na_V_1.7 using functional assays.

## 2. Results

### 2.1. OD1 Has Mixed α/β-Scorpion Toxin Activity at Na_V_1.7 at High Concentrations

**Figure 1:**
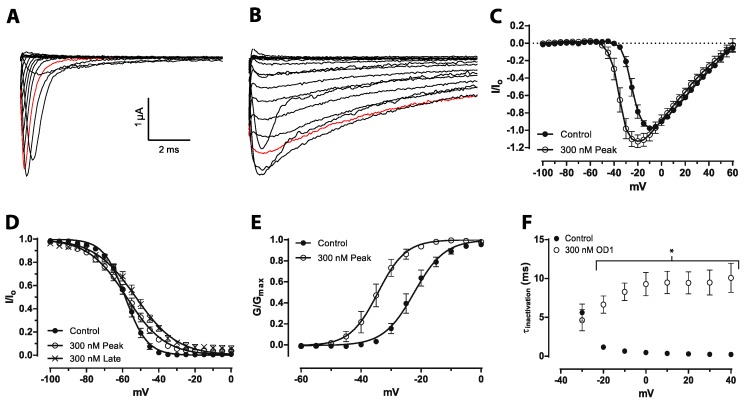
Activity of OD1 in CHO cells heterologously expressing hNa_V_1.7 assessed by automated patch clamping. Representative trace of sodium currents (**A**) before and (**B**) after addition of 300 nM OD1 elicited by depolarizing steps between −100 and +70 mV in 10-mV increments. The red trace highlights the depolarizing step to 0 mV. OD1 enhanced peak inward current and delayed inactivation, resulting in persistent current. (**C**) Current-voltage (IV) relationship before and after the addition of OD1 (300 nM). OD1 enhanced peak current with a leftward shift to more hyperpolarized potentials. (**D**) The voltage dependence of fast inactivation. OD1 significantly shifted the voltage dependence of fast inactivation for the late current 10 ms after depolarization (V_50_: control, −58.54 ± 0.23 mV; OD1_peak_, −57.78 ± 0.37; OD1_late_, −52.71 ± 0.49). (**E**) Voltage dependence of activation. OD1 shifted the voltage dependence of activation to a more hyperpolarized potential (V_50_: control, −22.47 ± 0.47; OD1 (300 nM), −34.50 ± 0.58). (**F**) Time of decay (*τ*) for inactivating current plotted *versus* test potential. OD1 (300 nM) delays fast inactivation at more depolarized membrane potentials. * *p* < 0.001 compared to the control. Data are presented as the mean ± SEM, *n* = 7.

OD1 was previously described as an α-scorpion toxin that enhances peak Na_V_1.7 current expressed in oocytes with a minor effect on the voltage dependence of channel activation or inactivation [14]. However, this effect is difficult to reconcile with the induction of spontaneous pain behavior *in vivo*, and we thus sought to evaluate the electrophysiological effects at Na_V_1.7 at concentrations that elicit robust spontaneous pain behaviors in mice (300 nM, see below). At a concentration of 300 nM, OD1 enhanced peak inward current and delayed inactivation, resulting in persistent current (Figure 1A,B), consistent with previously observed effects [14]. OD1 (300 nM) enhanced peak current with a leftward shift to more hyperpolarized potentials (Figure 1C). OD1 (300 nM) resulted in a small, non-significant shift in the V_50_ of inactivation for hNav1.7 peak current, while a significant shift in V_50_ of inactivation was observed for the late current 10 ms after depolarization (Figure 1D; V_50_ of inactivation: control, −58.54 ± 0.23 mV; OD1_peak_, −57.78 ± 0.37; OD1_late_, −52.71 ± 0.49). Although the V_50_ of inactivation is statistically unchanged at peak current, a significant fraction of hNav1.7 channels remain in the open state in the presence of OD1 (300 nM), even at conditioning potentials that completely inactivate untreated channels, as can be seen by the significantly increased slope of the voltage of inactivation (Figure 1D; *k*_h_ control = 6.07 ± 0.20 mV; *k*_h_ OD1_peak_ = 10.54 ± 0.33; *k*_h_ OD1_late_ = 11.45 ± 0.44; *p* < 0.05). Consistent with previous reports of mixed α/β toxin pharmacology on Na_V_1.4 and Na_V_1.6, a significant hyperpolarizing shift V_50_ of activation of −12 mV at Na_V_1.7 was observed in the presence of OD1 (300 nM) compared to control conditions (Figure 1E; V_50_ of activation: control, −22.47 ± 0.47; OD1, −34.50 ± 0.58; *p* < 0.05). OD1 (300 nM) also delayed fast inactivation at more depolarized membrane potentials (Figure 1F).

### 2.2. OD1 Causes Spontaneous Action Potential Firing in A- and C-Fibers

**Figure 2:**
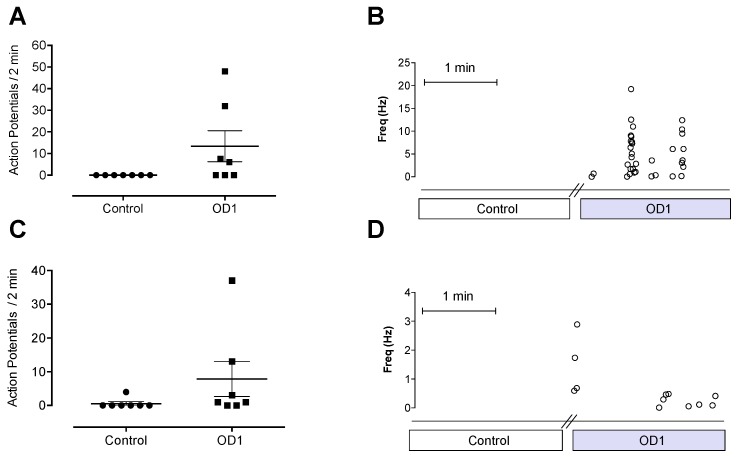
Effects of Na_V_1.7 activation by OD1 on A- and C-fibers using the mouse skin-saphenous nerve preparation. (**A**) OD1 caused spontaneous firing of action potentials in 57% of the A-fibers tested (action potentials/2 min: control, 0 ± 0; OD1 (30 nM), 13 ± 7; *n* = 7). (**B**) Action potentials plotted as a function of instantaneous firing frequency from an A-fiber before and after perfusion of OD1. Each point represents a single action potential, with the position on the *y*-axis indicating the frequency relative to the preceding action potential. (**C**) OD1 caused spontaneous firing of action potentials in 29% of the C-fibers tested (action potentials/2 min: control, 1 ± 0.6; OD1 (30 nM), 8 ± 5; *n* = 7). (**D**) Action potentials plotted as a function of instantaneous firing frequency from a C-fiber before and after perfusion of OD1. Each point represents a single action potential, with the position on the y-axis indicating the frequency relative to the preceding action potential.

To assess the effect of pharmacological Na_V_1.7 activation on A- and C-fibers, we tested OD1 using the mouse skin-saphenous nerve preparation. Consistent with the crucial role of Na_V_1.7 in regulating excitability, the application of OD1 to the receptive fields of peripheral sensory neurons led to spontaneous firing of action potentials in some fibers, with 57% of A-fibers tested (Figure 2A,B; control 0 ± 0 and OD1 (30 nM) 13 ± 7 action potentials/2 min; *n* = 7) and 29% of C-fibers tested firing spontaneously (Figure 2C,D; control 1 ± 0.6 and OD1 (30 nM) 8 ± 5 action potentials/2 min; *n* = 7) in the presence of OD1.

### 2.3. Intraplantar Injection of OD1 Causes Spontaneous Pain Behavior in Mice Mediated Through Na_V_1.7

**Figure 3:**
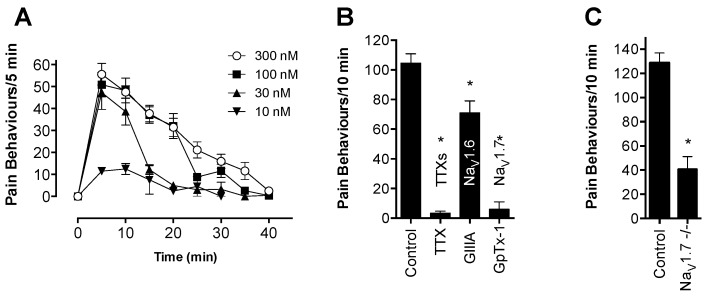
Mouse model of Na_V_1.7-mediated pain based on intraplantar injection of OD1. (**A**) Intraplantar injection of OD1 (10–300 nM) caused spontaneous pain behaviors, as evidenced by licking, flinching, lifting and shaking of the injected hind paw. Intraplantar injection of phosphate-buffered saline/0.1% BSA alone caused no spontaneous pain behaviors. (**B**) Intraplantar injection of TTX (1 μM) or the Na_V_1.7 inhibitor GpTx-1 (1 μM) completely reversed spontaneous pain behaviors, while the Na_V_1.6 inhibitor GIIIA (10 μM) only had a partial effect. (**C**) Na_V_1.7^−/−^ mice displayed significantly reduced spontaneous pain behaviors compared to Cre and loxP littermate controls. Statistical significance was determined using the *t*-test or one-way ANOVA with Dunnett’s post-test; * *p* < 0.05 compared to the control. Data are presented as the mean ± SEM, *n* = 3–9 per group.

To assess the effect of OD1 on pain behaviors in mice, we chose the intraplantar route of administration, as this route delivers the peptide directly to the terminals of peripheral sensory neurons in the skin, allowing simple quantification of unilateral pain behaviors while avoiding systemic adverse effects. Intraplantar injection of the Na_V_1.7 activator OD1 (10–300 nM) led to the development of spontaneous pain behaviors in a concentration-dependent manner, as evidenced by licking, flinching, lifting and shaking of the injected hind paw, consistent with the spontaneous action potential firing seen in A- and C-fibers (Figure 3A). These spontaneous pain behaviors developed rapidly, occurring immediately after injection, and persisted for up to 40 min after injection of the highest concentration (300 nM). Intraplantar injection of phosphate-buffered saline/0.1% BSA alone caused no spontaneous pain behaviors. No significant changes in mechanical (paw withdrawal force: OD1 (300 nM), 2.5 ± 0.5 g; control, 2.9 ± 0.5 g; *n* = 4, *p* > 0.05) or thermal thresholds (time to withdrawal: OD1 (300 nM), 8.0 ± 2 s; control, 11.0 ± 1 s; *n* = 4, *p* > 0.05) were detected 45 min after injection of OD1 (300 nM), the time by which spontaneous pain behaviors had resolved. As OD1 at a concentration of 300 nM was found to elicit robust responses, this concentration was used for further behavioral studies.

Since OD1 has activity at Na_V_1.6 at higher concentrations (EC_50_ = 30–47 nM) [15] and activation of Na_V_1.6 can also cause spontaneous pain behaviors [18], we next assessed the relative contribution of Na_V_1.7 and Na_V_1.6 to spontaneous pain behaviors induced by OD1 (Figure 3B). Co-administration of tetrodotoxin (TTX) with OD1 completely reversed spontaneous pain behaviors, confirming the involvement of only TTX-sensitive Na_V_ subtypes (spontaneous pain behaviors/10 min: control, 105 ± 6; TTX (1 μM), 3 ± 2); *p* < 0.05). Co-administration with the Na_V_1.6 inhibitor GIIIA [19] reduced spontaneous pain behaviors by 33%, indicating partial involvement of Na_V_1.6 (spontaneous pain behaviors/10 min: control, 105 ± 6; GIIIA (10 μM), 71 ± 8); *p* < 0.05). In contrast, co-administration with the Na_V_1.7 inhibitor GpTx-1 (see the full Na_V_ selectivity in Table 1) completely reversed spontaneous pain behaviors, confirming a major role for Na_V_1.7 in mediating spontaneous pain behaviors induced by OD1 (spontaneous pain behaviors/10 min: control, 105 ± 6; GpTx-1 (1 μM), 6 ± 5); *p* < 0.05). A major contribution of Na_V_1.7 to OD1-induced pain was also confirmed using Na_V_1.7^−/−^ mice, which displayed significantly reduced spontaneous pain behaviors compared to Cre and loxP littermate controls (Figure 3C; spontaneous pain behaviors/10 min: control, 129 ± 8; Na_V_1.7^−/−^, 41 ± 11; *p* < 0.05).

### 2.4. GpTx-1 is a Selective Na_V_1.7 Inhibitor that Reverses OD1-Induced Pain Behaviors

**Figure 4:**
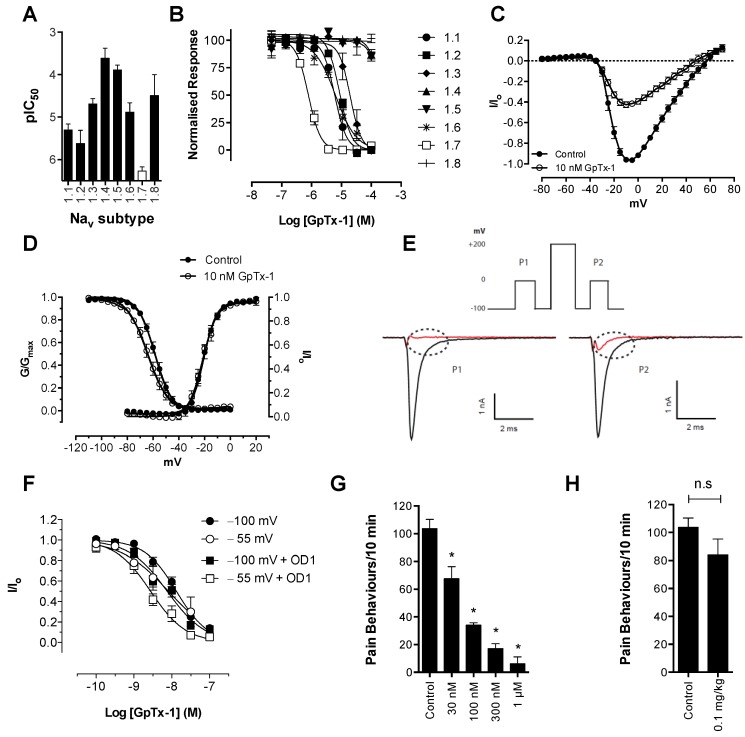
Na_V_ selectivity and *in vivo* effects of the spider peptide GpTx-1. Activity of GpTx-1 was assessed in HEK cells heterologously expressing Na_V_1.1–Na_V_1.8 using FLIPR membrane potential assays. (**A**) GpTx-1 selectively inhibited Na_V_1.7, with the following pIC_50_’s ranked in order of potency (hNa_V_1.7 (6.27 ± 0.1) > hNa_V_1.2 (5.63 ± 0.3) > hNa_V_1.1 (5.30 ± 0.1) > hNa_V_1.6 (4.88 ± 0.2) > hNa_V_1.3 (4.69 ± 0.1) > hNa_V_1.8 (4.49 ± 0.5) > hNa_V_1.5 (3.89 ± 0.1) > hNa_V_1.4 (3.61 ± 0.2). Data are presented as the mean ± SEM from three independent experiments. (**B**) Representative concentration response curves of GpTx-1 at Na_V_1.1–Na_V_1.8. (C–F) Activity of GpTx-1 in CHO cells heterologously expressing hNa_V_1.7 assessed by automated patch clamping. (**C**) Current-voltage (IV) relationship before and after the addition GpTx-1. (**D**) Voltage dependence of activation and fast inactivation before and after the addition of GpTx-1. GpTx-1 (10 nM) had no significant effect on the voltage of activation (V_50_: control, −20.52 ± 0.4 mV; GpTx-1 (10 nM), −21.4 ± 0.5 mV), but shifted the voltage dependence of fast inactivation to more hyperpolarized potentials (V_50_: control, −58.66 ± 0.3 mV; GpTx-1 (10 nM), −64.58 ± 0.5 mV). (**E**) Trace of sodium currents before (black) and after addition of 1 µM GpTx-1 (red). GpTx-1 causes full inhibition of the peak current that was partially reversed (highlighted by the points) by a strong depolarization pulse (+200 mV for 50 ms). (**F**) Concentration response curve of GpTx-1 elicited by a 20-ms pulse to 0 mV from a holding potential of −100 mV or an 8-s conditioning voltage step of −55 mV with and without OD1 (300 nM). GpTx-1 had no significant preference for the open/inactivated state (IC_50_ 8 nM) compared to the closed/resting state (IC_50_ 13 nM). The presence of OD1 (300 nM) did not significantly affect the potency of GpTx-1 in the open/inactivated state (IC_50_ 3 nM) or in the closed/resting state (IC_50_ 8 nM). Data are presented as the mean ± SEM, *n* = 10–14. (**G**) Intraplantar administration of GpTx-1 (30 nM, 100 nM, 300 nM, 1 μM) concentration-dependently reversed spontaneous pain behaviors in mice evoked by OD1. (**H**) Intraperitoneal administration of GpTx-1 (0.1 mg/kg) had no significant (n.s.; *p* > 0.05) effect on spontaneous pain behaviors in mice induced by OD1. Statistical significance was determined using the *t*-test or one-way ANOVA with Dunnett’s post-test; * *p* < 0.05 compared to the vehicle control. Data are presented as the mean ± SEM, *n* = 3–9 per group.

GpTx-1 was recently reported as a Na_V_1.7-selective inhibitor isolated from the venom of the tarantula *Grammostola porteri* (identical in sequence to GTx1-15 isolated from *Grammostola rosea* [20]); however, the mechanism of action of GpTx-1 and selectivity across all Na_V_ subtypes has not been reported. Using a Fluorometric Imaging Plate Reader (FLIPR) membrane potential assay, GpTx-1 was found to inhibit Na_V_1.7 with 100-fold selectivity over Na_V_1.4, Na_V_1.5 and Na_V_1.8, consistent with the selectivity profile previously reported from electrophysiological studies [9,20]. The GpTx-1 rank order of potency (pIC_50_ ± SEM) was hNa_V_1.7 (6.27 ± 0.1) > hNa_V_1.2 (5.63 ± 0.3) > hNa_V_1.1 (5.30 ± 0.1) > hNa_V_1.6 (4.88 ± 0.2) > hNa_V_1.3 (4.69 ± 0.1) > hNa_V_1.8 (4.49 ± 0.5) > hNa_V_1.5 (3.89 ± 0.1) > hNa_V_1.4 (3.61 ± 0.2) (Figure 4A,B; see also Table 1 for IC_50_ values and Figure S1 for a sample trace).

GpTx-1 inhibited the peak current of hNa_V_1.7 without significantly shifting the voltage dependence of activation (Figure 4C,D; V_50_: control, −20.52 ± 0.4 mV; GpTx-1 (10 nM), −21.4 ± 0.5 mV); however, a shift in the voltage dependence of fast inactivation to more hyperpolarized potentials was observed (Figure 4D; V_50_ for control −58.66 ± 0.3 mV and GpTx-1 (10 nM) −64.58 ± 0.5 mV). GpTx-1 caused full inhibition of peak current at 1 µM, which was partially reversed after a strong depolarizing pulse to +200 mV for 50 ms (Figure 4E), an effect often seen with gating modifier toxins [21]. GpTx-1 displayed minimal state dependence, with an IC_50_ of 8 nM in the open/inactivated state and 13 nM in the closed/resting state (Figure 4F). The potency of GpTx-1 was not significantly affected by the presence of OD1 (300 nM) in the open/inactivated state (IC_50_ 3 nM) or in the closed/resting state (IC_50_ 8 nM) (see Figure S2 for a sample trace).

**Figure 5:**
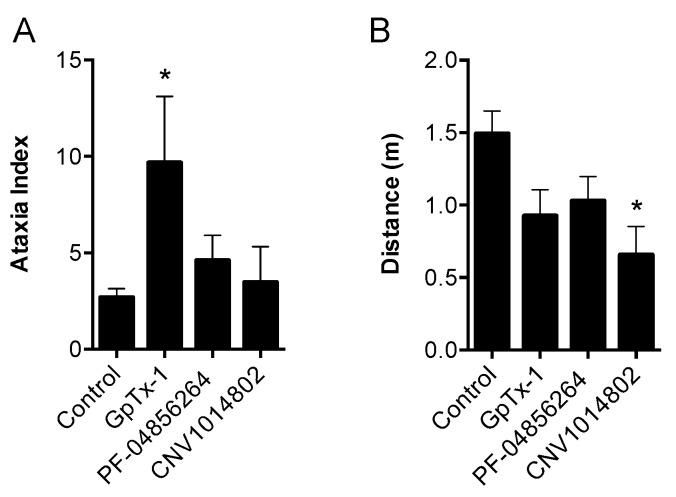
Motor assessment of systemically (i.p.) delivered GpTx-1, PF-04856264 and CNV1014802. (**A**) Ataxia index (number of foot slips per meters travelled) assessed by the Parallel Rod Floor Test. GpTx-1 (0.3 mg/kg; *n* = 4) significantly increased the ataxia index, while PF-04856264 (30 mg/kg; *n* = 5) and CNV1014802 (30 mg/kg; *n* = 3) had no significant effect compared to the vehicle control (*n* = 12). (**B**) Distance travelled (m) assessed by the Parallel Rod Floor Test. CNV1014802 (30 mg/kg; *n* = 3) significantly reduced the distance travelled, while GpTx-1 (0.3 mg/kg; *n* = 4) and PF-04856264 (30 mg/kg; *n* = 5) had no significant effect compared to the vehicle control (*n* = 12). Statistical significance was determined using one-way ANOVA with Dunnett’s post-test; * *p* < 0.05 compared to the vehicle control. Data are presented as the mean ± SEM.

Intraplantar injection of GpTx-1 co-administered with OD1 potently and concentration-dependently reduced spontaneous pain behaviors, causing almost complete reversal at the highest concentration (Figure 4G; spontaneous pain behaviors/10 min: control, 105 ± 6; GpTx-1 (1 μM), 6 ± 5; GpTx-1 (300 nM), 17 ± 4; GpTx-1 (100 nM), 34 ± 2; GpTx-1 (30 nM), 68 ± 9; *p* < 0.05). However, intraperitoneal injection of GpTx-1 at the highest tolerated dose (0.1 mg/kg) had no significant effect on spontaneous pain behaviors (Figure 4H). At a dose of 0.3 mg/kg, GpTx-1 causes motor deficits, as evidenced by a significant increase in the ataxia index (Figure 5A; ataxia index: control, 2.7 ± 0.4; GpTx-1 (0.3 mg/kg), 9.7 ± 3.4; *p* < 0.05). Therefore, it was not possible to assess the effect of GpTx-1 at systemic doses above 0.1 mg/kg.

### 2.5. PF-04856264 Is a Selective Na_V_1.7 Inhibitor that Reverses OD1-Induced Pain Behaviors

**Figure 6:**
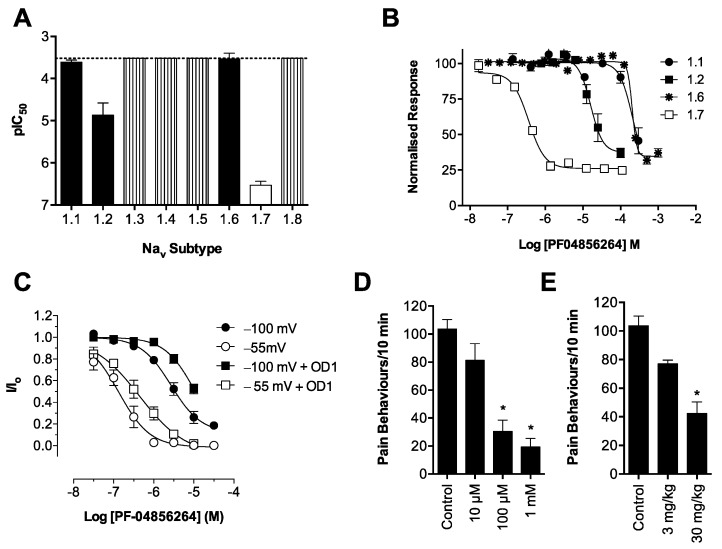
Na_V_ selectivity and *in vivo* effects of the aryl sulfonamide PF-04856264. Activity of PF-04856264 was assessed in HEK cells heterologously expressing Na_V_1.1–Na_V_1.8 using FLIPR membrane potential assays. (**A**) PF-04856264 selectively inhibited Na_V_1.7, with the following pIC_50_’s ranked in order of potency: hNa_V_1.7 (6.53 ± 0.1) > hNa_V_1.2 (4.87 ± 0.3) > hNa_V_1.1 (3.61 ± 0.1) > hNa_V_1.6 (3.53 ± 0.1). PF-04856264 had no inhibitory activity at the other Na_V_ subtypes up to a concentration of 300 μM. Data are presented as the mean ± SEM from three independent experiments. (**B**) Representative concentration response curves of PF-04856264 at Na_V_1.1, 1.2, 1.6 and 1.7. (**C**) Activity of PF-04856264 in CHO cells heterologously expressing hNa_V_1.7 assessed by automated patch clamping. Concentration response curve of PF-04856264 elicited by a 20-ms pulse to 0 mV from a holding potential of −100 mV or an 8 s conditioning voltage step of −55 mV with and without OD1 (300 nM). PF-04856264 preferentially bound to the open/inactivated state (IC_50_ 134 nM) compared to the closed/resting state (IC_50_ 2.7 μM). The presence of OD1 (300 nM) reduced the potency of PF-04856264 in both the open/inactive state (IC_50_ 445 nM) and the closed/resting state (IC_50_ 11 μM). Data are presented as the mean ± SEM, *n* = 6–14. (**D**) Intraplantar administration of PF-04856264 (10 μM, 100 μM, 1 mM) concentration-dependently reversed spontaneous pain behaviors in mice evoked by OD1. (**E**) Intraperitoneal administration of PF-04856264 (30 mg/kg) also significantly reduced spontaneous pain behaviors in mice. Statistical significance was determined using one-way ANOVA with Dunnett’s post-test; * *p* < 0.05 compared to the vehicle control. Data are presented as the mean ± SEM, *n* = 3–9 per group.

PF-04856264 is a novel aryl sulfonamide Na_V_1.7 inhibitor with little effect on Na_V_1.3 and Na_V_1.5, although activity at the other Na_V_ subtypes has not been reported [17]. Using the FLIPR membrane potential assay, we found that PF-04856264 was a selective Na_V_1.7 inhibitor with >40-fold selectivity over all other Na_V_ subtypes and >1000-fold selective over Na_V_1.4–Na_V_1.6. The rank order of potency (pIC_50_ ± SEM) was hNa_V_1.7 (6.53 ± 0.1) > hNa_V_1.2 (4.87 ± 0.3) > hNa_V_1.1 (3.61 ± 0.1) > hNa_V_1.6 (3.53 ± 0.1) >> hNa_V_1.3 ~ hNa_V_1.4 ~ hNa_V_1.5 ~ hNa_V_1.8 (Figure 6A,B; see also Table 1 for IC_50_ values and Figure S1 for a sample trace). Interestingly, PF-04856264 only partially inhibited responses in the FLIPR assay, leaving a residual 25%–30% change in membrane potential unaffected, despite completely inhibiting Na_V_1.7 current in patch clamp assays (see below). PF-04856264 acts as a gating modifier, interacting with the voltage sensor in Domain IV and preferentially binding to and stabilizing the inactivated state of Na_V_1.7 [17]. We confirmed the state dependence of PF-04856264 inhibition, finding a 20-fold shift in the Na_V_1.7 IC_50_ from closed/resting state inhibition (2.7 μM) to open/inactivated state inhibition (134 nM) (Figure 6C). Interestingly, in the presence of OD1 (300 nM), PF-04856264 had a three-fold loss of potency in both the closed/resting state (IC_50_ 11 μM) and the open/inactivated state (IC_50_ 445 nM) (see Figure S2 for a sample trace).

Intraplantar injection of PF-04856264 concentration-dependently reduced spontaneous pain behaviors induced by co-administered OD1 (300 nM), although surprisingly, the high local concentrations required did not correlate with its *in vitro* potency (Figure 6D; spontaneous pain behaviors/10 min: control, 105 ± 6; PF-04856264 (1 mM), 19 ± 6; PF-04856264 (100 μM), 31 ± 8; PF-04856264 (10 μM), 81 ± 12; *p* < 0.05). Intraperitoneal administration of PF-04856264 was also effective (Figure 6E; spontaneous pain behaviors/10 min: control, 105 ± 6; PF-04856264 (30 mg/kg), 42 ± 8; *p* < 0.05) and well tolerated, with no significant motor adverse effects observed at doses up to 30 mg/kg, correlating well with the *in vitro* selectivity profile (Figure 5A; ataxia index: control, 2.7 ± 0.4; PF-04856264 (30 mg/kg), 4.6 ± 1.3; *p* > 0.05; Figure 5B; distance travelled: control, 1.49 ± 0.16 m; PF-04856264 (30 mg/kg), 1.03 ± 0.17 m; *p* > 0.05).

### 2.6. CNV1014802 is a Non-Selective Na_V_ Inhibitor

**Figure 7:**
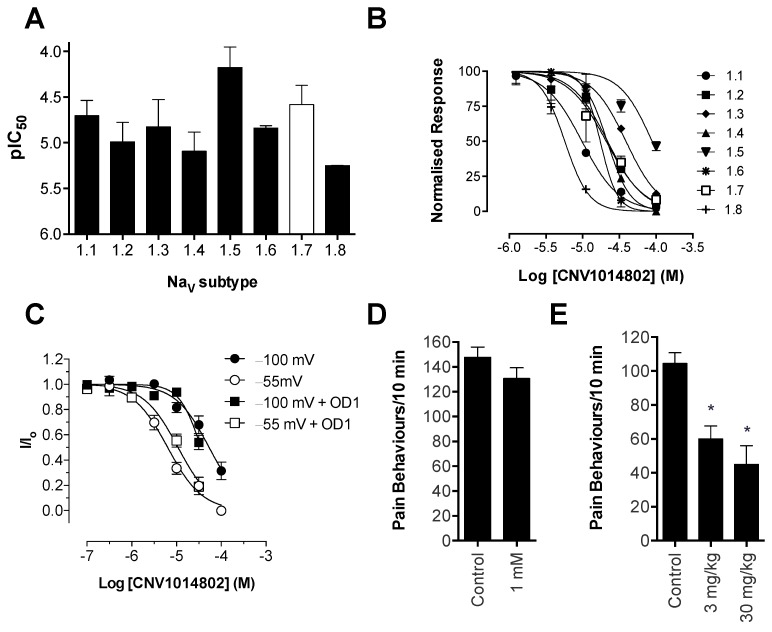
Na_V_ selectivity and *in vivo* effects of the clinical candidate CNV1014802. Activity of CNV1014802 was assessed in HEK cells heterologously expressing Na_V_1.1–Na_V_1.8 using FLIPR membrane potential assays. (**A**) CNV1014802 non-selectively inhibited Na_V_ channels, with the following pIC_50_’s ranked in order of potency: hNa_V_1.8 (5.25 ± 0.1) > hNa_V_1.4 (5.09 ± 0.2) > hNa_V_1.2 (4.99 ± 0.2) > hNa_V_1.6 (4.84 ± 0.1) > hNa_V_1.3 (4.82 ± 0.3) > hNa_V_1.1 (4.70 ± 0.2) > hNa_V_1.7 (4.58 ± 0.2) > hNa_V_1.5 (4.18 ± 0.2). Data are presented as the mean ± SEM from three independent experiments. (**B**) Representative concentration response curves of CNV1014802 at Na_V_1.1–1.8. (**C**) Activity of CNV1014802 in CHO cells heterologously expressing hNa_V_1.7 assessed by automated patch clamping. Concentration response curve of CNV1014802 elicited by a 20-ms pulse to 0 mV from a holding potential of −100 mV or an 8-s condition voltage step of −55 mV with and without OD1 (300 nM). CNV1014802 preferentially bound to the open/inactivated state (IC_50_ 6.3 μM) compared to the closed/resting state (IC_50_ 54 μM). The presence of OD1 (300 nM) did not significantly affect the potency of CNV1014802. Data are presented as the mean ± SEM, *n* = 4–7. (**D**) Intraplantar administration of CNV1014802 (1 mM) had no significant effect on spontaneous pain behaviors in mice evoked by OD1. (**E**) However, intraperitoneal administration of CNV1014802 (3 and 30 mg/kg) significantly reduced spontaneous pain behaviors in mice. Statistical significance was determined using the *t*-test or one-way ANOVA with Dunnett’s post-test; * *p* < 0.05 compared to the vehicle control. Data are presented as the mean ± SEM, *n* = 3–9 per group.

CNV1014802 is reported to be a state-dependent inhibitor of Na_V_1.7; however, information on the potency and selectivity profile has not been reported [8]. Surprisingly, CNV1014802 was found to be a relatively non-selective Na_V_ inhibitor, in the FLIPR membrane potential assay, with a rank order of potency (pIC_50_ ± SEM): hNa_V_1.8 (5.25 ± 0.1) > hNa_V_1.4 (5.09 ± 0.2) > hNa_V_1.2 (4.99 ± 0.2) > hNa_V_1.6 (4.84 ± 0.1) > hNa_V_1.3 (4.82 ± 0.3) > hNa_V_1.1 (4.70 ± 0.2) > hNa_V_1.7 (4.58 ± 0.2) > hNa_V_1.5 (4.18 ± 0.2) (Figure 7A,B; see also Table 1 for IC_50_ values and Figure S1 for a sample trace). We were able to confirm the state-dependence reported for CNV1014802, finding a nine-fold shift in the Na_V_1.7 IC_50_ between closed/resting state inhibition (54 μM) and open/inactivated state inhibition (6.3 μM), with a preference for the open/inactivated state (Figure 7C). Compared to PF-04856264, the potency of CNV1014802 at Na_V_1.7 is 50-fold weaker; however, the presence of OD1 (300 nM) did not significantly affect the potency of CNV1014802 at Nav1.7 in the closed/resting state (IC_50_ 35 μM) or the open/inactivated state (IC_50_ 11 μM) (see Figure S2 for a sample trace).

Despite *in vitro* activity at hNa_V_1.7, CNV1014802 was unable to significantly attenuate OD1-induced spontaneous pain behaviors when delivered locally by intraplantar injection at a concentration of 1 mM (Figure 7D). However, CNV1014802 was effective when delivered by the intraperitoneal route (Figure 7E; spontaneous pain behaviors/10 min: control, 105 ± 6; CNV1014802 (30 mg/kg), 45 ± 5; CNV1014802 (3 mg/kg), 60 ± 8; *p* < 0.05), with sedative adverse effects observed at the highest dose, as evidenced by a significant reduction in distance travelled, but no significant increase in the ataxia index (Figure 5A; ataxia index: control, 2.7 ± 0.4; CNV1014802 (30 mg/kg), 3.5 ± 1.9; *p* > 0.05; Figure 5B; distance travelled: control, 1.49 ± 0.16 m; CNV1014802 (30 mg/kg), 0.66 ± 0.20 m; *p* < 0.05).

## 3. Discussion

Choosing a relevant animal pain model to evaluate the analgesic efficacy of Na_V_1.7-selective inhibitors is an essential step in the pre-clinical development of these compounds. We therefore developed a novel mouse model of Na_V_1.7-mediated pain based on intraplantar injection of the scorpion toxin OD1 that allows rapid *in vivo* characterization of the analgesic efficacy of Na_V_1.7 inhibitors.

OD1 was initially classed as a α-scorpion toxin that increases peak current by delaying fast inactivation, but has little effect on the voltage dependence of activation at Na_V_1.7 expressed in oocytes at 50 nM. [14,22]. Similarly, a small, albeit statistically-significant shift in the voltage dependence of activation was recently reported at Na_V_1.7 expressed in mammalian cells [23]. Our results confirm that this shift in the voltage dependence of activation is further enhanced at higher concentrations. Thus, OD1 has mixed α/β-scorpion toxin effects at Na_V_1.7, not only increasing peak current and delaying inactivation, but also shifting the voltage dependence of activation to more hyperpolarized potentials. Thus, the mixed functional activity of OD1, which was also previously reported for Na_V_1.4 and Na_V_1.6 [15], appears to be concentration dependent, suggesting that OD1 preferentially interacts with the α-scorpion toxin-binding site with additional interaction with the β-scorpion toxin-binding site at higher concentrations. Both pharmacological effects are consistent with the ability of OD1 to induce spontaneous action potential firing in sensory neurons. While pure α-toxins would be expected to amplify supra-threshold responses due to the inhibition of channel inactivation and would lead to amplification of small generator potentials, a shift in the voltage dependence of activation would additionally result in channel opening closer to resting membrane potential, resulting in greater amplification of sub-threshold responses and induction of spontaneous action potential firing.

The functional effects of OD1 on Na_V_1.7 can be correlated to the functional consequences of Na_V_1.7 gain-of-function mutations in the painful syndromes inherited erythromelalgia and paroxysmal extreme pain disorder. Typically, mutations present in inherited erythromelalgia patients cause a voltage-dependent shift of activation of Na_V_1.7 to more hyperpolarized potentials [24,25,26,27], corresponding to the β-scorpion toxin-like effects of OD1, whereas paroxysmal extreme pain disorder mutations generally delay the fast inactivation [28,29,30], corresponding to the α-scorpion toxin activity of OD1. This indicates that the OD1 mouse model may be used to identify new analgesics with the potential to treat the poorly managed conditions of inherited erythromelalgia and paroxysmal extreme pain disorder.

Na_V_1.7 is preferentially expressed in the majority of peripheral sensory neurons that have been functionally characterized as nociceptive A- and C-fibers [31]. Specifically, it is present on the peripheral terminals of nociceptive neurons, where it contributes to the amplification of sub-threshold generator potentials by producing ramp currents that depolarize the membrane to the threshold required to generate an action potential [32,33]. Application of OD1 to the receptive fields of A- and C-fibers caused spontaneous action potential firing, consistent with the effect of OD1 on the biophysical properties of Na_V_1.7, which would result in depolarization closer to resting membrane potential, leading to spontaneous action potential firing. The spontaneous pain behaviors induced by intraplantar injection of OD1 are consistent with the spontaneous action potential firing seen in A- and C-fibers in the skin-nerve preparation.

Although OD1 is a selective Na_V_1.7 activator, it also has activity at Na_V_1.6, albeit at higher concentrations. Given that intraplantar administration of the Na_V_1.6 activator Cn2 also causes spontaneous pain behaviors in mice [18], we assessed the relative contribution of Na_V_1.7 and Na_V_1.6 to spontaneous pain behaviors induced by OD1. While the Na_V_1.6 inhibitor GIIIA partially reversed OD1-induced spontaneous pain by 33%, the Na_V_1.7 selective inhibitors GpTx-1 and PF-04856264 were effective after intraplantar injection at concentrations unlikely to significantly inhibit Na_V_1.6 *in vivo*, suggesting that the majority of spontaneous pain behaviors induced by OD1 are mediated through Na_V_1.7. This was further confirmed in Na_V_1.7^−/−^ mice, where the behavioral phenotype was strongly attenuated, with residual responses likely attributable to the Na_V_1.6 activity of OD1. Thus, OD1 elicited a robust and long-lasting behavioral response that was mediated predominantly through Na_V_1.7, allowing the efficacy of Na_V_1.7 inhibitors to be readily assessed for their potential in the treatment of Na_V_1.7-mediated pain.

Consistent with its reported activity at Na_V_1.7 [20], GpTx-1 was found to significantly reduce OD1-induced spontaneous pain behaviors when administered locally. The reasons for the potent *in vivo* effects of GpTx-1 are not entirely clear. Peptide Na_V_ inhibitors can have particularly slow off-rates, which could contribute to high apparent *in vivo* potency. In addition, it is currently unclear what level of Na_V_1.7 inhibition is required to cause analgesia, although it is reasonable to assume that complete inhibition of Na_V_1.7 current may not be required to prevent Na_V_1.7-mediated action potential firing. Thus, for compounds that are able to achieve relevant free drug concentrations at peripheral nerve endings and that can achieve full inhibition of current at relevant states, the *in vivo* IC_50_ may be lower than the observed *in vitro* IC_50_. Although effective locally, we were not able to reproduce this effect with systemic delivery of GpTx-1. This is likely due to dose-limiting side effects, as the maximum tolerated systemic dose (0.1 mg/kg) may not have achieved sufficiently high concentration at peripheral nerve endings in the hind paw to inhibit Na_V_1.7. Motor adverse effects were observed at doses above 0.1 mg/kg, limiting administration of higher doses, and were most likely attributable to activity at Na_V_1.6, which is expressed in the periphery on sensory and motor neurons, where loss of function leads to movement disorders and hind limb paralysis [34]. This suggests that greater than the 30-fold selectivity for Na_V_1.7 over Na_V_1.6 seen with GpTx-1 is required to have an acceptable therapeutic window for systemic use; however, off-target activity at other Na_V_ isoforms may also contribute to the adverse effects.

In contrast, the aryl sulfonamide PF-04856264 displayed excellent selectivity (>1000 fold) for Na_V_1.7 over Na_V_1.4–Na_V_1.6. This selectivity profile was reflected *in vivo*, with relatively high systemic doses (30 mg/kg) causing no significant motor adverse effects. PF-04856264 was effective at reversing OD1-induced spontaneous pain behaviors when delivered both locally and systemically, although higher than expected doses were required to elicit analgesia by either routes relative to its potency in the FLIPR membrane potential (IC_50_ 300 nM) and electrophysiology (IC_50_ 130 nM) assays. However, given that PF-04856264 preferentially binds to Na_V_1.7 in the inactivated state, it is possible that the delayed inactivation produced by OD1 promoted an unfavorable channel state for PF-04856264 binding. Indeed, the presence of OD1 led to a three-fold loss of potency in the electrophysiological assays. State-dependent inhibition of Na_V_1.7 may also explain the inability of PF-04856264 to fully block membrane potential changes using the fluorescence assay, where residual responses could be mediated by veratridine-modified channels resistant to inactivation that in turn are unaffected by PF-04856264 [35]. The physiological relevance of a preference for the inactivated state of Na_V_1.7 in other pathological pain conditions remains to be established, although such a mechanism might imply lack of efficacy in paroxysmal extreme pain disorder, where Na_V_1.7 channels are relatively resistant to fast inactivation.

Consistent with previous reports, CNV1014802 also was a state-dependent inhibitor of Na_V_1.7 in our hands, although it lacked the Na_V_ subtype selectivity of PF-04856264. Surprisingly, CNV1014802 lacked *in vivo* analgesic efficacy when delivered locally at a concentration of 1 mM, perhaps reflecting its relatively poor potency at Na_V_1.7 in membrane potential (32 μM) and electrophysiology (6.3 μM) assays. In addition, CNV1014802 (formally GSK1014802 and now raxatrigine) is reported to be highly (94%) protein bound, which could also lead to a decrease in free effective drug at the site of action after local administration [36]. In contrast to local administration, CNV1014802 was effective when administered systemically, although some sedative adverse effects were observed at the highest dose (30 mg/kg). These effects are consistent with the reported ability of CNV1014802 to penetrate the central nervous system, which likely is crucial for its efficacy in animal models of seizures and schizophrenia [36,37], suggesting that analgesic activity after systemic dosing may also be due to central effects.

Based on our pharmacological validation, the OD1-induced spontaneous pain model can be used to assess the *in vivo* activity of compounds when delivered by both the intraplantar and a systemic route. An advantage of the intraplantar route is that it uses considerably less material and thus may be the preferable route of administration for screening compounds for *in vivo* activity in the early stages of lead identification. However, as was the case for PF-04856264 and CNV1014802, *in vivo* activity in the OD1 model did not correspond well with the *in vitro* potency found in the electrophysiology and FLIPR membrane potential assays. At least in the case of PF-04856264, species-specific effects are unlikely to contribute to this discrepancy, as equipotency at human and mouse Na_V_1.7 has been previously reported [17]. In addition, although PF-04856264 lost potency in the presence of OD1, consistent with preferential binding to the inactivated state, this change was relatively small and would not explain the relative lack of potency *in vivo*. Interestingly, we observed incomplete inhibition of Na_V_1.7-mediated responses in the FLIPR membrane potential assay by PF-04856264 and CNV1014802 (Figure S1), although both compounds completely inhibited currents from Na_V_1.7 patch-clamp recordings. While it is unclear whether this effect correlates to the ability of these compounds to prevent activity-induced membrane depolarization at peripheral sensory nerve endings *in vivo*, such effects may contribute to the poor *in vivo* activity observed. In addition, it is plausible that other factors, such as the pharmacokinetic properties of these compounds, affect *in vivo* efficacy. Nonetheless, these results highlight the need for a simple animal model, such as the OD1 model described here, which permits rapid evaluation of *in vivo* on-target effects, as the *in vitro* properties do not always correspond well to *in vivo* activity.

Scorpion α-toxins, such as OD1, are known to bind to Domain IV [38]; however, we have demonstrated that efficacy in the OD1 model is not dependent on competitive binding at this site. For example, TTX, which binds to the pore of the channel, and GpTx-1, which binds to Domain II [39], were both able to completely abolish OD1-induced spontaneous pain behaviors. Although not as potent *in vivo*, the state-dependent inhibitors PF-04856264, predicted to share an overlapping binding site with scorpion α-toxins on Domain IV [17], and CNV1014802 (binding site remains to be determined) were also effective. Thus, the OD1 model is suitable to evaluate *in vivo* activity of Na_V_1.7 inhibitors irrespective of their mechanism of action or binding site.

In conclusion, we have established a Na_V_1.7-mediated mouse model of pain based on intraplantar injection of the scorpion toxin OD1 and pharmacologically characterized the activity of the spider peptide GpTx-1, the small molecule PF-04856264 and the clinical candidate CNV1014802. The model is suitable to evaluate *in vivo* on-target activity of Na_V_1.7 inhibitors; however, it is unclear if activity in OD1-induced pain translates to anti-nociceptive or anti-allodynic effects in other rodent models or analgesia in humans. While efficacy in the OD1 model is useful to validate *in vivo* on-target activity at Na_V_1.7, species differences need to be considered carefully when using animal models of human disease. Given the relatively high homology between mouse and human Na_V_ isoforms, significant pharmacological differences have rarely been reported. Indeed, PF-04856264 is equipotent at mouse and human Na_V_1.7 [17], and the effects of OD1 on rodent Na_V_1.7 closely match the effects on human Na_V_1.7 [15]. While Na_V_1.7 is arguably a well-validated pain target in humans based on genetic evidence from loss-of-function and gain-of-function conditions, including congenital insensitivity to pain, erythromelalgia and paroxysmal extreme pain disorder, it remains to be established if pharmacological inhibition of Na_V_1.7 can achieve global graded analgesia.

## 4. Experimental Section

### 4.1. Chemicals

OD1 was synthesized as previously described [15]. PF-04856264 was from SYNthesis Med Chem (Parkville, VIC, Australia) and CNV1014802 was from Axon MedChem (Groningon, The Netherlands). GIIIA was synthesized by Boc chemistry using methods previously described [40]. TTX was from Tocris Bioscience (Bristol, UK), and veratridine was from Alomone Labs (Jerusalem, Israel). All other reagents were from Sigma Aldrich (Castle Hill, NSW, Australia), unless otherwise stated. Peptides were routinely diluted in 0.1% bovine serum albumin (BSA).

### 4.2. Synthesis of GpTx-1

Solvents for reversed-phase high-performance liquid chromatography (HPLC) consisted of 0.05% trifluoroacetic acid (TFA)/H_2_O (A) and 90% acetonitrile/0.043% TFA/H_2_O (B). Analytical HPLC was performed on a Shimadzu LC20AT system (Shimadzu, Kyoto, Japan) using a Thermo Hypersil GOLD 2.1 × 100 mm C18 column (ThermoScientific, Brendale, QLD, Australia) heated at 40 °C with flow rate of 0.3 mL/min. A gradient of 10–55% B over 30 min was used, with detection at 214 nm. Preparative HPLC was performed on a Vydac 218TP1022 column (Grace, Columbia, MD, USA) running at a flow rate of 16 mL/min using a gradient of 5%–45% B over 40 min. Mass spectrometry was performed on an API2000 (ABI Sciex, Mt Waverley, VIC, Australia) mass spectrometer in positive ion mode.

Protected 9-fluorenylmethyloxycarbonyl (Fmoc) amino acid derivatives and 2-(1*H*-benzotriazol-1-yl)-1,1,3,3-tetramethyluronium hexafluorophosphate (HBTU) were purchased from Iris Biotech (Marktredwitz, Germany); Rink-amide resin, N,N-dimethylformamide (DMF), *N*,*N*-diisopropylethylamine (DIEA) and TFA from Auspep (Tullamarine, VIC, Australia). All other reagents were obtained from Sigma-Aldrich (Castle Hill, NSW, Australia).

GpTx-1 was assembled on a Symphony (Protein Technologies Inc., Tucson, AZ, USA) automated peptide synthesizer on a Rink-amide (loading 0.67 mmol/g) polystyrene resin on a 0.1 mmol scale. Fmoc deprotections were achieved using 30% piperidine/DMF (1 × 1.5 min, then 1 × 4 min). Couplings were performed in DMF using 5 equivalents of Fmoc-amino acid/HBTU/DIEA (1:1:1) relative to resin loading for 2 × 20 min. Amino acid side-chains were protected as Asn(Trt), Asp(OtBu), Arg(Pbf), Cys(Trt), His(Trt), Lys(Boc), Ser(tBu), Thr(tBu), Trp(Boc), Tyr(tBu). Cleavage from the resin and removal of side-chain protecting groups was achieved by treatment with 95% TFA/2.5% TIPS/2.5% H_2_O at room temperature for 2 h. After most of the cleavage solution was evaporated under a stream of N_2_, the product was precipitated and washed with cold Et_2_O and lyophilized from 50% acetonitrile/0.1% TFA/H_2_O 223 mg; ESI-MS (*m/z*): calc. (average) 1021.0 [M + 4H]^4+^, found 1020.8.

Purified reduced peptide (41 mg), reduced glutathione (100 equiv) and oxidized glutathione (10 equiv.) were dissolved in 6 M GnHCl (21 mL) then added to a solution of 0.36 M NH_4_OAc (pH 8.0, 230 mL) and stirred at room temperature with exposure to air for 48 h. The single major product was isolated by preparative HPLC: 13.5 mg; ESI-MS (*m/z*): calc. (average) 1019.5 [M + 4H]^4+^, found 1019.4. The crude product was purified by preparative HPLC to give 42 mg of hexathiol GpTx-1 (99% purity).

### 4.3. Animals

Ethical approval for *in vivo* experiments in animals was obtained from The University of Queensland animal ethics committee (PHARM/261/13/ARC and IMB/PACE/326/15). Experiments involving animals were conducted in accordance with the Animal Care and Protection Regulation Qld (2012), the Australian Code of Practice for the Care and Use of Animals for Scientific Purposes, 8th edition (2013, and the International Association for the Study of Pain Guidelines for the Use of Animals in Research. All efforts were made to minimize animal suffering and to reduce the number of animals used.

For behavioral assessment, we used adult male C57BL/6J mice aged 6–8 weeks. Animals were housed in groups of 3 or 4 per cage, under 12-h light-dark cycles and had standard rodent chow and water *ad libitum*. Advillin-Cre constitutive Na_V_1.7 knockout mice were kindly provided by Professor John N. Wood (University College London) [41].

### 4.4. Behavioral Assessment Following Injection of OD1

OD1 was diluted in phosphate-buffered saline/0.1% BSA (10–300 nM) and administered by shallow subcutaneous (intraplantar) injection to the left hind paw of mice in a volume of 40 μL under light isoflurane (3%) anesthesia. Mice were then placed individually into polyvinyl boxes (10 × 10 × 10 cm), and spontaneous pain behavior (licks and flinches) was counted by an investigator unaware of the treatments received from video recordings for 40 min post-injection at 5-min intervals. Once spontaneous pain behavior had ceased, mechanical and thermal allodynia was assessed using an electronic von Frey apparatus (MouseMet Electronic von Frey, TopCat Metrology, Ely, UK) and the Hargreaves method (Plantar Analgesia Meter, IITC, Woodland Hills, CA, USA), as previously described [42].

### 4.5. Effect of Pharmacological Inhibitors and Clinical Compounds

OD1 (300 nM) was administered as described above, and cumulative spontaneous pain behaviors were quantified for 10 min immediately post-injection of OD1 by a blinded observer. Compounds delivered by the intraplantar route were co-injected with OD1 at the concentrations stated. Compounds delivered by the intraperitoneal route were administered 15 min prior to injection of OD1 at the doses stated in a volume of 10 μL/g. All compounds were diluted in phosphate-buffered saline, except PF-04856264 and CNV1014802, which were diluted in phosphate-buffered saline/10% DMSO due to poor solubility.

### 4.6. Motor Performance Assessment

Motor performance was assessed using the Parallel Rod Floor Test and analyzed using ANY-Maze software (Stoelting Co., version 4.70, Wood Dale, IL, USA). GpTx-1 (0.3 mg/kg), PF-04856264 (30 mg/kg) and CNV1014802 (30 mg/kg) were administered by the intraperitoneal route, as described above, 15 min prior to assessment of motor performance. Mice were then placed in the Parallel Rod Floor Test apparatus, and the distance travelled (m) and number of foot slips were recorded over 1 min using the ANY-Maze software. The ataxia index was calculated by dividing the number of foot slips by the distance travelled (m).

### 4.7. Skin-Nerve Preparation

The effect of OD1 on A- and C-fibers was assessed using single fiber recordings from isolated mouse skin-saphenous nerve preparations, as previously described [43]. Briefly, the saphenous nerve along with the skin of the dorsal hind paw and lower leg of adult C57BL/6J mice was removed and placed in an organ chamber, perfused with carbogenated synthetic interstitial fluid containing the following (in mM): NaCl (107.8), KCl (3.5), MgSO_4_ (0.69), NaHCO_3_ (26.2), NaH_2_PO_4_ (1.67), Na-gluconate (9.64), glucose (5.55), sucrose (7.6) and CaCl_2_ (1.53); pH 7.3. The saphenous nerve was placed in a separate recording chamber immersed in paraffin oil and was desheathed and teased apart until a single fiber recording was identified by mechanically probing the corresponding receptive field. Fibers were then classified based on conduction velocity assessed by electrical stimulation of the receptive field with a microelectrode (C-fiber < 1 m/s, A-fiber 1.6–12 m/s). The receptive field was isolated using a plastic ring, and OD1 (30 nM) was continuously perfused at a rate of 8 mL/min at 32 °C. Data were recorded and analyzed using DAPSYS, Version 8.

### 4.8. Cell Culture

Human embryonic kidney (HEK 293) cells stably expressing hNa_V_1.1, hNa_V_1.2, hNa_V_1.3, hNa_V_1.4, hNa_V_1.5, hNa_V_1.7 and hNa_V_1.8 (SB Drug Discovery, Glasgow, UK) were cultured in MEM containing 10% *v/v* fetal bovine serum supplemented with L-glutamine (2 mM) and selection antibiotics as recommended by the manufacturer. HEK 293 cells stably expressing hNa_V_1.6 (kind gift from Dr. Frank Lehmann-Horn, University of Ulm, Germany) were cultured in DMEM containing 10% *v/v* fetal bovine serum and geneticin 0.5 mg/mL. CHO cells stably expressing hNav1.7 (Chantest, OH, USA) were cultured in Ham’s F12 containing 10% *v/v* fetal bovine serum and selection antibiotics, as recommended by the manufacturer. Cells were grown in a humidified 5% CO_2_ incubator at 37 °C, grown to 70%–80% confluence and passaged every 3–4 days using TrypLE Express (Invitrogen, Scoresby, VIC, Australia).

### 4.9. FLIPR Membrane Potential Assays

HEK 293 cells stably expressing hNa_V_1.1–1.8 were plated 48 h before the assay on 384-well black-walled imaging plates at a density of 10,000–15,000 cells per well and were loaded with red membrane potential dye (Molecular Devices, San Francisco, CA, USA) according to the manufacturer’s instructions for 30 min at 37 °C. After the addition of GpTx-1, PF-04856264 and CNV1014802 using the FLIPR^TETRA^, cells were incubated a further 5 min before stimulating Na_V_ using veratridine (60 μM, Na_V_1.1–Na_V_1.7) or deltamethrin (150 μM, Na_V_1.8). Changes in membrane potential were assessed using the FLIPR^TETRA^ (excitation 515–545 nm, emission 565–625 nm) every second for 300 s after adding agonists. To quantitate the effect of test compounds on Na_V_ responses, the area under the curve (AUC) corresponding to 300 s after the addition of veratridine was computed using ScreenWorks (Molecular Devices, Version 3.2.0.14) and normalized to buffer responses (0%) and veratridine control responses (100%).

### 4.10. Electrophysiology

CHO cells were used for the electrophysiology, because in our experience, they are more robust to clamp compared to HEK cells. CHO hNa_V_1.7 were passaged 48 h prior to the assay in a T-175 flask and cultured in selection-free media at 37 °C or CHO hNav1.7 EZCells™ were thawed the day of the experiment according to the manufacture’s specifications. To induce expression in the CHO hNa_V_1.7 cells, tetracycline (1 μg/mL) was added to culture media 24 h prior to the assay. Cells were harvested at 70%–80% confluence and resuspended to 1 × 10^6^ cells/mL in Ex-Cell ACF CHO Medium with 25 mM HEPES and 1× Glutamax (Gibco, Thermo Fisher Scientic, Scoresby, VIC, Australia) before being transferred to the QPatch QStirrer and allowed to recover for 30 min. Extracellular solution (EC) was used to resuspend all test compounds and contained (in mM): NaCl (140), KCl (4), CaCl_2_ (2), MgCl_2_ (1), HEPES (10), CdCl_2_ (0.1) and glucose (10). The pH was adjusted to 7.4 with NaOH, and osmolarity was adjusted with sucrose to 315 mOsm. The intracellular solution contained (in mM): CsF (140), EGTA/CsOH (1/5), HEPES (10) and NaCl (10). The pH was adjusted to 7.4 with CsOH and osmolarity adjusted with sucrose to 320 mOsm. OD1 and GpTx-1 were diluted in EC with the addition of 0.1% BSA.

Whole-cell patch-clamp experiments were performed at room temperature on a QPatch-16 automated electrophysiology platform (Sophion Bioscience, Ballerup, Denmark) using 16-channel planar patch chip plates (QPlates; Sophion Bioscience) with a patch hole diameter of 1 µm and a resistance of 2 ± 0.02 MΩ. Cell positioning and sealing parameters were set as follows: positioning pressure −60 mbar, minimum seal resistance 0.1 GΩ, holding potential −100 mV, holding pressure −20 mbar. Whole-cell currents were filtered at 5 kHz and acquired at 25 kHz.

Cells expressing hNa_V_1.7 were maintained with a holding potential of −100 mV between voltage protocols. Current-voltage (IV) relationships were determined using a family of 500-ms conditioning pulses from −120 mV to +70 mV in 5-mV steps, followed by depolarization to 0 mV to assess the voltage dependence of fast inactivation (repetition interval: 20 s). Both peak and late current (10 ms post-peak) of the IV family were measured. State-dependence was assessed after a 10-min compound incubation (±OD1 300 nM) to ensure steady-state inhibition for each concentration. In order to assess compound activity at the partially inactivated/open state, a 50-ms depolarization to 0 mV was measured after a conditioning pulse to −55 mV for 8 s, with a 50-ms recovery period between (repetition interval 12 s). To assess possible interaction with the voltage sensor domain, a triple-pulse protocol was used, comprising two steps to 0 mV for 50 ms separated by a strong depolarization step to +200 mV for 50 ms with 20-ms recovery to the −100-mV holding potential between each step.

### 4.11. Data Analysis and Statistics

Data were plotted and analyzed using GraphPad Prism (GraphPad Softare, Version 6.0, La Jolla, CA, USA). Statistical significance was defined as *p* < 0.05 and was determined by the *t*-test or one-way ANOVA with Dunnett’s post-test, as appropriate. Data are expressed as the mean ± standard error of the mean. For electrophysiology experiments, the voltage dependence of steady-state inactivation was calculated by dividing the amplitude of the test current (*I*) by the maximal current elicited (*I_o_*), as defined by: *I_Na_ = I/I_o_*. The voltage-dependence of activation was derived from IV curves after normalizing for Na^+^ conductance (*G_Na_*) using: *G_Na_ = I_Na_/(V_memb_ − V_rev_)*, where *V_memb_* is the membrane potential and *V_rev_* is the reversal potential. Steady-state kinetic parameters were obtained by fitting the data to Boltzmann equations *y* = bottom + (top − bottom)/(1 + *exp*((V_50_ − *x*)/slope)). Sophion QPatch Assay Software (Sophion, Version 5.0, Ballerup, Denmark) was used to determine the time of decay (τ) for inactivating current using the equation for first-order exponential decay: *y = y_0_ × exp(- k × x) + y_p_*, where *y* is current, *x* is time, *y_0_* is peak current, *y_p_* is the plateau and *k* is the rate constant. τ was calculated separately for each conditioning potential, defined as 1/*k.*

## Figures and Tables

**Table 1:** IC_50_ values (in μM) at hNa_V_1.1–1.8 obtained from the Fluorometric Imaging Plate Reader (FLIPR) membrane potential assay.

Na_V_ Subtype	GpTx-1	PF-04856264	CNV1014802
1.1	6 ± 2	247 ± 27	22 ± 7
1.2	5 ± 2	20 ± 11	13 ± 6
1.3	22 ± 6	>300	23 ± 13
1.4	326 ± 170	>300	10 ± 5
1.5	140 ± 40	>300	84 ± 34
1.6	17 ± 9	309 ± 91	15 ± 1
1.7	0.58 ± 0.1	0.30 ± 0.07	32 ± 14
1.8	68 ± 32	>300	6 ± 0.2

## Supplementary Materials

The following are available online at www.mdpi.com/2072-6651/8/3/78/s1.
