# Supplementary material for: Analgesic Effects of GpTx-1, PF-04856264 and CNV1014802 in a Mouse Model of NaV1.7-Mediated Pain
Source: Toxins (Basel). 2016 Mar 17;8(3):78. doi: 10.3390/toxins8030078 (PMC4810223; doi:10.3390/toxins8030078)
Supplement: Supplementary file 1 [file toxins-08-00078-s001.pdf]

Analgesic Effects of GpTx-1, PF-04856264 and CNV1014802 in a Mouse Model of Nav1.7-Mediated Pain

Jennifer R. Deuis, Joshua S. Wingerd, Zoltan Winter, Thomas Durek, Zoltan Dekan, Silmara R. Sousa, Katharina Zimmermann, Tali Hoffmann, Christian Weidner, Mohammed A. Nassar, Paul F. Alewood, Richard J. Lewis, Irina Vetter

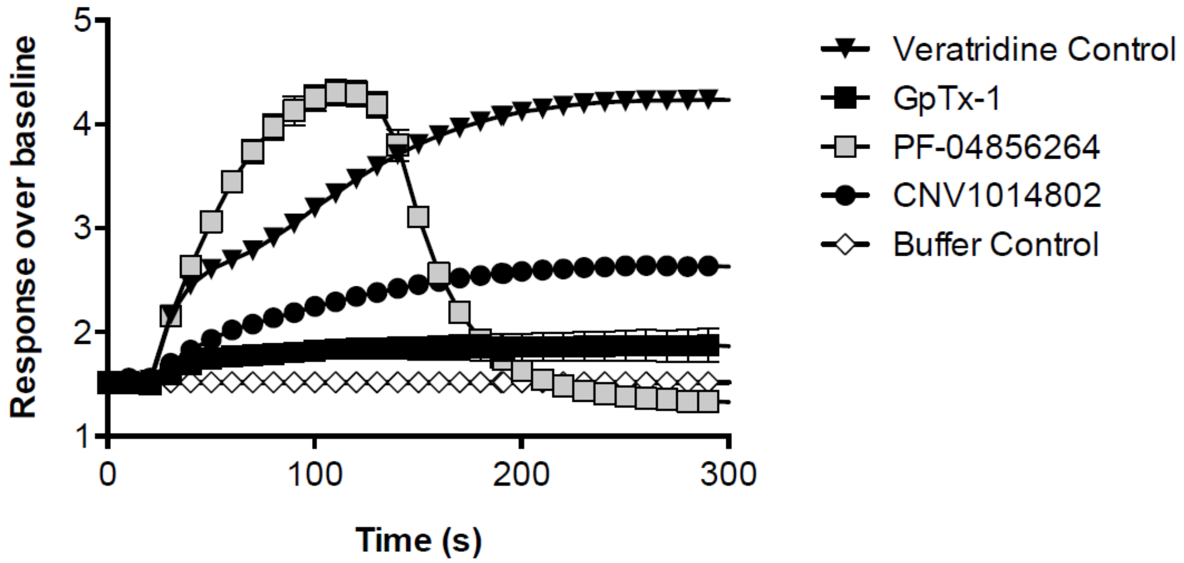

Figure S1. Sample trace of the FLIPR membrane potential assay in HEK cells heterologously expressing Nav1.7. GpTx-1 (100 μM), PF-04856264 (10 μM) and CNV1014802 (100 μM) all inhibited the veratridine-induced response. Response over baseline every 10 s is shown for clarity. Data is presented as mean ± SEM, *n* = 4–12 wells.

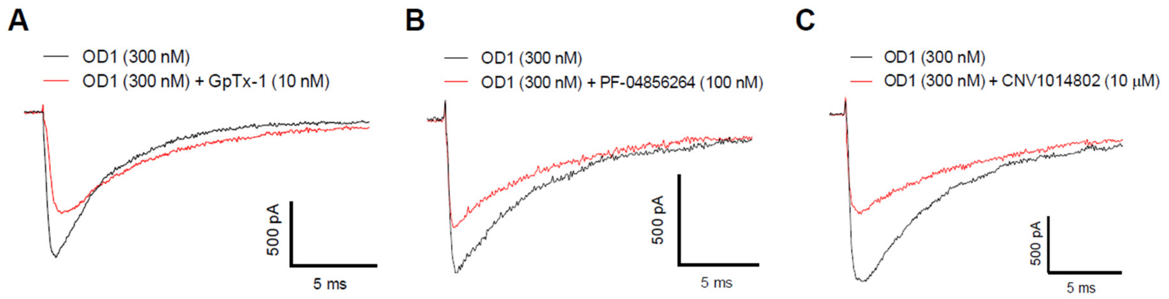

Figure S2. Sample current traces from CHO cells heterologously expressing hNav1.7 assessed by automated patch clamping. Currents were elicted by a 20 ms pulse to 0 mV with an 8 s conditioning voltage step of −55 mV in the presence of OD1 (300 nM) ± compounds. (**A**) GpTx-1; (**B**) PF-04856264 and (**C**) CNV1014802 all inhibited peak current, but did not affect OD1-induced persistent current.
